# Interaction of porcine reproductive and respiratory syndrome virus proteins with SUMO-conjugating enzyme reveals the SUMOylation of nucleocapsid protein

**DOI:** 10.1371/journal.pone.0189191

**Published:** 2017-12-13

**Authors:** Cong Wang, Nanfang Zeng, Siyu Liu, Qi Miao, Lei Zhou, Xinna Ge, Jun Han, Xin Guo, Hanchun Yang

**Affiliations:** Key Laboratory of Animal Epidemiology of the Ministry of Agriculture, College of Veterinary Medicine and State Key Laboratory of Agrobiotechnology, China Agricultural University, Beijing, People’s Republic of China; Nanjing Agricultural University, CHINA

## Abstract

SUMOylation is a reversible post-translational modification that regulates the function of target protein. In this study, we first predicted by software that the multiple proteins of porcine reproductive and respiratory syndrome virus (PRRSV) could be sumoylated. Next, we confirmed that Nsp1β, Nsp4, Nsp9, Nsp10 and nucleocapsid (N) protein of PRRSV could interact with the sole SUMO E2 conjugating enzyme Ubc9, and Ubc9 could be co-localized with Nsp1β, Nsp4, Nsp9 and Nsp10 in the cytoplasm, while with N protein in both the cytoplasm and nucleus. Finally, we demonstrated that N protein could be sumoylated by either SUMO1 or SUMO2/3. In addition, the overexpression of Ubc9 could inhibit viral genomic replication at early period of PRRSV infection and the knockdown of Ubc9 by siRNA could promote the virus replication. These findings reveal the SUMOylation property of PRRSV N protein and the involvement of Ubc9 in PRRSV replication through interaction with multiple proteins of PRRSV. To our knowledge, this is the first study indicating the interplay between SUMO modification system and PRRSV.

## Introduction

Porcine reproductive and respiratory syndrome virus (PRRSV) is considered as an intractable pathogen for swine production, causing great economical losses to global swine industry [1–3]. The virus was first characterized in Europe in 1991 and in the US in 1992 independently [4, 5]. Although numerous efforts have been made to control clinical diseases caused by PRRSV infection, PRRSV remains endemic in many countries worldwide [6–9]. PRRSV is an enveloped, single-stranded, positive-sense RNA virus, belonging to the genus *Arterivirus* of family *Arteriviridae* in the order *Nidovirales*, together with equine arteritis virus (EAV), lactate dehydrogenase-elevating virus (LDV) and simian hemorrhagic fever virus (SHFV) [10]. The whole genome of PRRSV is approximately 15 kb in length, and contains at least 12 overlapping open reading frames (ORFs) [11–13]. The ORF1a and ORF1b encode two large polyproteins (pp1a and pp1ab), which can be processed into at least 16 viral nonstructural proteins (nsps) including Nsp1α, Nsp1β, Nsp2, Nsp2TF, Nsp2N, Nsp3, Nsp4, Nsp5, Nsp6, Nsp7α, Nsp7β, Nsp8, and Nsp9-12 [14–19]. These nsps are shown to play important roles in replication and transcription of viral genome, and in modulating the host innate immunity, and in pathogenesis and virulence [13, 15, 20–22]. The remaining ORFs encode the structural proteins of PRRSV, including GP2a, E, GP3, GP4, GP5, M, N and ORF5a [23–25]. Recently, the Nsp2 has been recognized to be an integral membrane protein of PRRSV [21, 26].

Post-translational modification (PTM), including phosphorylation, glycosylation, acetylation, ubiquitination and SUMOylation etc, is one of the crucial mechanisms of regulating the function of proteins. Meanwhile, PTM affects a variety of biological processes of cells, such as cellular differentiation and development, and rapid and specific responses to endogenous or exogenous stimuli. SUMOylation is recognized a process of isopeptide bond formation between the carboxyl group of the modifier and the ε-amino group of a lysine residue in the target protein, and considered to be a major regulatory system of protein function that targets many substrates through direct SUMOylation and protein-protein interactions [27]. The modifier is named small ubiquitin-like modifier (SUMO), with the molecular mass of approximately 12 kDa. In vertebrate, SUMO shares four isoforms (SUMO1 to SUMO4). Of them, SUMO1 is quite divergent with only 47% identity with SUMO2 and SUMO3 which share 97% identity to each other and cannot be distinguished by antibodies, and however, the newly identified SUMO4 with a unique amino acid (Pro90), cannot be conjugated to target proteins [28, 29]. Generally, a SUMOylation amino acid consensus motif ψ-Lys-Xaa-Asp/Glu recognizes the matured SUMO proteins via a three-step enzymatic cascade [30]. The single SUMO-activating enzyme E1, which consists of a heterodimer of the Aos1/SAE1 and Uba2/SAE2 proteins, activates and transfers the proteolytic matured SUMO to the conjugating enzyme E2 Ubc9. Ubc9 is one of the Class I family members of E2 enzymes [31], and its specific transfer is well-characterized function of transferring SUMO paralogs to substrates with equal efficiency [32]. Of the large number of the ubiquitin E2, Ubc9 is the sole SUMO-conjugating enzyme acting as a hub for protein SUMOylation although SUMOylation is not detectable in all the Ubc9-interacting proteins [33]. The transfer of the SUMO from Ubc9 to the target protein is often facilitated by several SUMO ligases (e.g. PIAS family and RanBP2) [30]. A small family of SUMO isopeptides, especially the sentrin-specific proteases (SENPs), plays an important role in the reversion of this process [34].

The diverse cellular processes can be modulated by SUMOylation [27, 35]. Viruses have evolved to hijack the conserved SUMOylation system for their own benefits. Recent advances have suggested that varieties of viral proteins serve as the targets of the SUMOylation machinery by ways of affecting either their intracellular localization or their biological functions [36, 37]. Moreover, many viral proteins participate in the regulation of host SUMOylation system by not only interacting with SUMO pathway proteins but also mimicking the related enzymes [36–38]. Notably, quite a lot of proteins of either DNA or RNA viruses can interact with Ubc9, the hub of protein SUMOylation, leading to the modification of host protein status or the SUMOylation of the viral proteins [36, 37]. Mainly through directly recognizing and SUMOylating the Lys residues embedded in a SUMOylation consensus motif (SCM) by Ubc9, the target proteins can be sumoylated [39–41]. It is an effective way to search for the potential SUMO-targets of viral proteins through prediction of the consensus sites and interaction with Ubc9. Despite of numerous viral proteins being recognized as SUMO-targets, to date, little is known about the relationship between PRRSV and SUMOylation system.

In this study, we analyzed the amino acid sequences of PRRSV structural and nonstructural proteins and found that many conserved lysine residues in various proteins of PRRSV were predicted to be sumoylated based on similarity to the ψKxE consensus motif and to other well-known SUMOylation sites. Subsequently, we investigated the evidence that the Nsp1β, Nsp4, Nsp9, Nsp10 and N protein of PRRSV interact directly with the only conjugating enzyme Ubc9, and further predicted the potentiality of these proteins to be sumoylated. Moreover, we characterized the SUMOylation form of N protein during PRRSV infection.

## Materials and methods

### Ethics statement

The animal used for preparation of porcine pulmonary alveolar macrophages (PAMs) in this study was approved by The Laboratory Animal Ethical Committee of China Agricultural University. The animal treatment was performed according to the Chinese Regulations of Laboratory Animals—The Guidelines for the Care of Laboratory Animals (Ministry of Science and Technology of People’s Republic of China), and Laboratory Animal-Requirements of Environment and Housing Facilities (GB 14925–2010, National Laboratory Animal Standardization Technical Committee).

### Animals

Healthy piglets (Landrace) were obtained at the age of six weeks from Beijing Center for SPF Swine Breeding & Management that is known to be free of PRRSV. Prior to the sacrifice by euthanasia for preparation of PAMs, the animals were housed in the animal facilities at China Agricultural University (CAU) for two days, and were given access to food and water *ad libitum*.

### Cells, virus and infection

Preparation of PAMs from piglets was performed as previously described [42]. PAMs were then maintained in RPMI-1640 medium (Fisher Scientific, Waltham, MA) with 10% fetal bovine serum (FBS, Hyclone Laboratories, Inc., South Logan, UT). The African green monkey kidney epithelial cell line MARC-145 cells (ATCC) [43], and human embryonic kidney (HEK) 293 cells [44] and 293FT cells (Cell Resource Center, Institute of Basic Medical Science, CAMS/PUMC) [45] were cultured with GIBCO Dulbecco’s modified Eagle medium (DMEM) (Fisher Scientific) with 10% FBS in a 37°C incubator with 5% CO_2_. The PRRSV (JXwn06) used in this study was characterized in our laboratory [46]. For virus infection assays, MARC-145 cells were cultured to approximately 90% confluence and infected with JXwn06 at a multiplicity of infection (MOI) of 0.01, and then maintained in the medium containing 5% FBS at 37°C until collection.

### Antibodies and chemicals

The mouse anti-HA (Sigma-Aldrich, St. Louis, MO), anti-GFP (Proteintech, Chicago, IL), anti-GST (Abbkine, Redlands, CA), anti-SUMO1 (Fisher Scientific) and anti-SUMO2/3 (Abcam, Cambridge, UK) monoclonal antibody (mAb), and rabbit anti-Myc and anti-Ubc9 (Sigma-Aldrich) polyclonal antibody (PAb) were purchased commercially. The mouse anti-Nsp1β, Nsp4, Nsp9 and Nsp10 mAb were prepared in our laboratory. The mouse anti-N protein mAb was kindly given by Ping Jiang (Nanjing Agriculture University, Nanjing, China). 4’, 6-diamidino-2-pheny-lindol (DAPI) (Beyotime, Shanghai, China), N-Ethylmaleimide (NEM) (Sigma-Aldrich), Iodoracetamide (IAA) and protease inhibitor cocktail (Amresco, Solon, OH) were used in this study.

### Plasmid construction

The cDNA encoding Ubc9 was amplified from porcine pulmonary alveolar macrophages (PAMs) by RT-PCR with the designed primers (Table 1) based on the sequence of porcine Ubc9 gene available in GenBank (accession no: NM_001204369). The amplified product was confirmed by sequencing and then subcloned into the respective vectors, including pCMV-Myc and pGADT7 (Clontech Laboratories Inc., Mountain View, CA), pWPXL and pGEX-6p-1 (Addegene, Cambridge, MA). All the pGBKT7-bait plasmids (Clontech) were prepared in our laboratory, including individual plasmid that was expressing each nonstructural (except for Nsp2TF, Nsp2N, Nsp6 and Nsp8) and structural proteins of PRRSV JXwn06. The Nsp1β, Nsp4, Nsp9, Nsp10 and N gene of PRRSV JXwn06 was amplified by PCR using the plasmid pWSK-JXwn06 as template [46] and each fragment was cloned into pCMV-HA (Clontech). Meanwhile, the N gene was cloned into the plasmid pGEX-6p-1.

**Table 1:** Primers used in this study.

Primer^a^	Sequence(5’-3’)^b^	Product length (bp)	Use
1F	AGGGACTGGAGATGTCGG	581	Ubc9 amplification
1R	GATTGCAGAGAGCAACGTG		
2F	CGGAATTCATGTCGGGGATCGCCCTC (EcoRI)	501	Ubc9 amplification and clone into pCMV-Myc
2R	ATAGTTTAGCGGCCGCTTATGAGGGAGCAAACTTCTTGG(NotI)		
3F	CGGGATCCAATGTCGGGGATCGCCCTCAG(BamHI)	493	Ubc9 amplification and clone into pWPXL
3R	CGACGCGTAATGAGGGAGCAAACTTCTTGGCTTG(MluI)		
4F	CGGAATTCATGTCGGGGATCGCCCTCC(EcoRI)	495	Ubc9 amplification and clone into pGADT7
4R	CCGCTCGAGCTTATGAGGGAGCAAACTTC(XhoI)		
5F	CCGGAATTCATGTCGGGGATCGCC(EcoRI)	495	Ubc9 amplification and clone into pGEX6p-1
5R	CCGCTCGAGTTATGAGGGAGCAAACTTC(XhoI)		
6F	CCGGAATTCATGCCAAATAACAACG(EcoRI)	390	N amplification and clone into pGEX6p-1
6R	CCGCTCGAGTCATGCTGAGGGTGAT(XhoI)		
7F	CCAGCCAGTCAATCAGCTG*A*GCCAAATGCTGG		Mutagenesis of N (C23S)
7R	C*T*CAGCTGATTGACTGGCTGGCCATTCCCCTT		
8F	CCAGCCAGTCAATCAGCTG*GC*CCAAATGCTGG		Mutagenesis of N (C23A)
8R	C*CG*AGCTGATTGACTGGCTGGCCATTCCCCTT		

^a^ F denotes forward PCR primer; R denotes reverse PCR primer.

^b^ Restriction sites are underlined. Mutated nucleotides are shown as italics.

### Preparation of Nsp1β-, Nsp4-, Nsp9-, Nsp10-, N- and Ubc9-expressing lentiviruses

A lentiviral packaging system, including pWPXL (Cat No. 12257), pMD2.G (Cat No. 12259) and psPAX2 (Cat No. 12260), was purchased from Addgene (Addegene, Cambridge, MA, USA). The lentivirus-mediated protein expression was performed according to the standard protocol. Briefly, the fragment of N and Ubc9 gene was subcloned into the expression vector pWPXL, and the other plasmids, pWPXL-Nsp1β, pWPXL-Nsp4, pWPXL-Nsp9 and pWPXL-Nsp10, were prepared in our laboratory [47]. Each construct was mixed with pMD2.G and psPAX2 in the appropriate proportion, and then co-transfected into the HEK 293FT cells of 80% confluence by using the FuGENE^®^ HD Transfection reagents (Promega, Madison, WI). When a number of syncytia appeared, the supernatants containing lentiviruses were harvested and filtered with a 0.45 filter (Pall Corporation, Port Washington, NY) to remove cell debris, and then concentrated by Amicon ultra-100 centrifuge tubes (Mick Millipore, Billerica, MA). The titers of lentiviruses were determined using a QuickTiter Lentivirus Titer Kit (Lentivirus-associated HIV p24) (Cell Biolabs, San Diego, CA) and the lentiviruses were stored for use at -80°C. Then respective proteins were expressed in MARC-145 cells that transduced with the lentiviruses in the presence of 8 μg/mL polybrene (Sigma). At 24 h post-transduction, the inoculums were removed and the cells were maintained in DMEM containing 10% FBS.

### Yeast two-hybrid screening for protein interaction

The yeast two-hybrid assay was carried out according to the manufacturer’s instruction. Briefly, by co-transforming of the respective bait (pGBKT7) and prey (pGADT7) plasmids into the yeast strain Y2HGold (Clontech), the co-transformants were selected on high-stringency quadruple dropout (QDO) plates containing 0.07 μg/ml aureobasidin A (ABA) and 0.04 mg/ml 5-bromo-4-chloro-3-indoyl-α-D-galactopyranoside (X-α-Gal) (Clontech).

### Western blot analysis

For Western blot and immunoprecipitation (IP), the cells grown in plates were washed three times with pre-cooled PBS, detached by scraping into cell lysis buffer (Beyotime) containing NEM, IAA and a complete protease inhibitor cocktail. The cells were lysed for 30 min with gentle rotation and the insoluble material was removed by centrifugation at 12, 000 g for 15 min at 4°C. SDS-PAGE and Western blot were carried out following standard methods. Briefly, the proteins in cell lysates were separated on 12% polyacrylamide gel by SDS-PAGE, the proteins were transferred onto polyvinylidene difluoride membranes (PVDF) (Sigma), which were then blocked with 5% skim milk powder in PBS for 2 h at room temperature (RT). The membranes were washed with PBST and incubated with respective primary antibody diluted in the primary antibody diluents (TOYOBO, Japan) according to the manufacturer’s instruction overnight at 4°C. Thereafter, a secondary HRP-labeled goat anti-mouse IgG (ZB-2305) or goat anti-rabbit IgG (ZB-2301) (ZSGB-BIO, Beijing, China) diluted at 1:10000 in the secondary antibody diluents was added to the membranes and incubated for 1 h at 37°C. Subsequently, the membrane was washed three times with TBST and examined with a chemiluminescence detection kit (Fisher, Scientific) and finally exposed to a chemiluminescence apparatus (ProteinSimple, Santa Clara, CA).

### Co-immunoprecipitation assay

The cells were harvested and lysed in pre-cooled Co-IP lysis buffer at 36 h post-transfection. The cell lysates were precipitated with a proposed mAb-conjugated Protein A sepharase (GE Healthcare, Fairfield, CT) overnight at 4°C with gentle rotation. The beads were washed five times with 1 ml of the lysis buffer and then boiled with 5×SDS loading buffer (Beyotime) for 5 min. The supernatants were subjected to SDS-PAGE analysis.

### Protein expression, purification, and GST pull-down assay

The pGEX-6p-1 and pGEX-6p-1-Ubc9 were transformed in *Escherichia coli* strain BL21, respectively, and the GST or GST-Ubc9 proteins were induced with 1mM isopropyl-β-D-thiogalactopyranoside (IPTG). The cells were harvested at 12 h post-induction, and resuspended in pre-cooled PBS, and homogenized by sonication. The lysates were cleared by centrifugation, and the supernatant was subjected to Sepharose 4B-glutathione resin (GE Healthcare) for affinity purification. GST-Ubc9 proteins were eluted from the column with 10 mM glutathione elution buffer.

For GST pull-down assay, equal amounts of purified GST or GST-Ubc9 proteins were bound to glutathione agarose (Fisher Scientific), respectively, according to the manufacturer’s instruction and the beads were washed four times using PBS. The recombinant HA-tagged Nsp4, Nsp9, Nsp10 or N protein was harvested from transfected HEK293 cells individually, and incubated with pull-down lysis buffer for 2 h at 4°C. The eluted proteins were detected by SDS-PAGE and Western blot.

### Confocal imaging

For the co-localization analysis of exogenous Ubc9 with the proteins of PRRSV, HEK293 cells grown on coverslips in 24-well plates at 70 ~ 80% confluence were co-transfected with pCMV-Myc-Ubc9 and each HA-tagged protein of PRRSV using the Lipofectmine LTX and PLUS reagents. At 36 h post-transfection, the cells were fixed with 100% pre-cooled ethyl alcohol for 15 min at RT, and washed with PBS for three times, and then incubated with the mixture of anti-HA mAb and anti-Myc PAb for 1 h at 37°C. After three times wash with PBS, the cells were incubated with TRITC-conjugated goat anti-mouse IgG or FITC-conjugated goat anti-rabbit IgG for 1 h at 37°C. Then the nuclear DNA was stained with DAPI for 4 min. The images were obtained with a Nikon TE-2000E or Olympus confocal microscope.

For the co-localization analysis of endogenous Ubc9 with the proteins of PRRSV, MARC-145 cells and PAMs were infected with PRRSV JXwn06 at a multiplicity of infection (MOI) of 0.01. At 36 h post-infection, the cells were fixed with 100% pre-cooled ethyl alcohol, and then washed with PBS. The cells were probed with an anti-Nsp4, Nsp9, Nsp10 or N mAb, and an anti-Ubc9 PAb, followed by TRITC-conjugated goat anti-mouse IgG or FITC-conjugated goat anti-rabbit IgG for 1 h at 37°C. After three washes with PBS, the nuclei were stained with DAPI for 4 min at RT. The treated cells were then visualized under confocal microscope.

### PRRSV infection in MARC-145 cells

MARC-145 cells grown up to 80% confluence in 10 cm plates were infected with PRRSV JXwn06 at a MOI of 0.01 in DMEM with 5% FBS. At 48 h post-infection, the cell lysates were subjected to Co-IP assay with corresponding antibody, and the lysates of mock-infected cells were chosen as a control.

### SiRNA-mediated *Ubc9* knockdown in MARC-145 cells

The siRNAs to porcine Ubc9 gene silencing were synthesized (GenePharma, Shanghai, China). When MARC-145 cells were grown to 80% confluence in 6-well plates, 30 pmol of Ubc9 siRNA was transfected with the Lipofectamine RNAiMax (Fisher Scientific) according to the manufacturer’s protocol. The cell lysates were subjected to Western blot analysis at 48 h post-transfection. The siRNA sequences targeting Ubc9 were as follows: siubc9-1(sense), 5’-GAGGCCUACACGAUUUACUTT-3’; siubc9-2 (sense), 5’-GCACGAUGAACCUCAUGAATT-3’; siubc9-3 (sense), 5’-CCAUCACAAUCAAACAGAUTT-3’; the control siCon (sense), 5’-UUGCGGGUCUAAUCACCGATT-3’.

### PRRSV infection in Ubc9-overexpressed MARC-145 cells

MARC-145 cells in 24-well culture plates were transduced with the lentiviruses that were expressing Ubc9. At 24 h post-transduction, the cells were washed three times with PBS and then infected with PRRSV JXwn06 at a MOI of 0.01. Then the infected cells were washed with PBS after incubation for 1 h and maintained in DMEM containing 5% FBS. The cells were harvested at 12 h, 24 h, 36 h, 48 h, 60 h and 72 h post-infection, both the culture supernatants and the cells were collected for virus titers analysis [46], and the cells infected with PRRSV were collected for qRT-PCR. Similarly, the Ubc9-silenced MARC-145 cells were infected with the virus as described above.

### Quantitative RT-PCR assay

TRIzol reagent (Fisher Scientific) was used to extract the viral RNA from cellular samples. The cDNA was reverse-transcribed from 1 μg of total RNAs using a Quant One Step RT-PCR Kit (TIANGEN, Beijing, China). The primers for detecting the mRNA levels of PRRSV N protein gene and internal control β-actin gene were designed as follows: forward 5’-AATAACAACGGCAAGCAGCAA-3’ and reverse 5’-CCTCTGGACTGGTTTTGTTGG-3’ for N gene; forward 5’-CTCCATCATGAAGTGCGACGT-3’ and 5’-GTGATCTCCTTCTGCATCCTGTC-3’ for β-actin gene. The procedure for quantitative RT-PCR was carried out as described previously [48].

### Drug treatment

SUMOylation inhibitor, Ginkgolic acid (GA) (Sigma-Aldrich) was diluted in pure methanol (Me) at the stock concentration of 1mM. The MARC-145 cells were treated with GA for 4 h at a working concentration of 50 μM and Me was served as a control. Subsequently, the cells were infected with PRRSV JXwn06 at a MOI of 0.01 for 1 h. Then the infected cells were washed with PBS and maintained in DMEM containing 5% FBS. The cells were harvested at 12 h, 24 h, 36 h, 48 h, 60 h and 72 h post-infection, the virus titers were measured [46].

### Statistical analysis

The data presents means ± standard deviations (SD). The Graphpad Prism software (version 5.0) was used to determine the significance of the variability among different groups by Two-way ANOVA test of variance. A *p* < 0.05 was considered to be statistically significant.

## Results

### In silico analysis predicts multiple SUMOylation sites in PRRSV proteins

The electron-rich and nucleophilic nature of the lysine side chain makes it suitable for covalent post-translational modification with diverse substrates, including ubiquitination, SUMOylation and acetylation. For instance, the lysine residues embedded in the SUMOylation consensus motif (ψ-Lys-Xaa-Asp/Glu) can be potentially modulated by the SUMOylation system, thus three SUMO prediction algorithms, including SUMOsp2.0^a^ [49], SUMOplot^™^ (http://www.abgent.com/sumoplot.html) and seeSUMO [50], were applied to predict the potential consensus motifs and SUMO acceptors site in structural and nonstructural proteins of PRRSV. The proteins of PRRSV which were predicted to contain multiple SCMs are shown in Table 2.

**Table 2:** Predicted SUMOylation sites in the nonstructural and structural proteins of PRRSV.

Proteins of PRRSV	SUMOSp1.0^a^	SUMOplot	seeSUMO
Nsp1α	M^b^	L	Y
Nsp1β	H	H	Y
Nsp2	H	H	Y
Nsp3	-	-	Y
Nsp4	M	H	Y
Nsp5	-	-	Y
Nsp6	L	-	Y
Nsp7	L	H	-
Nsp8	-	-	Y
Nsp9	H	H	Y
Nsp10	L	-	Y
Nsp11	L	H	Y
Nsp12	-	-	Y
ORF2a	-	-	Y
ORF2b	-	-	Y
ORF3	-	H	Y
ORF4	-	L	Y
ORF5	-	H	Y
ORF6	H	H	Y
ORF7	-	L	Y

^a^ Three web servers for sequence-based prediction of protein sumoylation sites.

^b^ H, M, L and Y represents High, Medium, Low threshold and YES respectively; —means undetected.

### PRRSV Nsp1β, Nsp4, Nsp9, Nsp10 and N proteins are the targets of Ubc9 by a yeast-two hybrid assay

**Fig 1:**
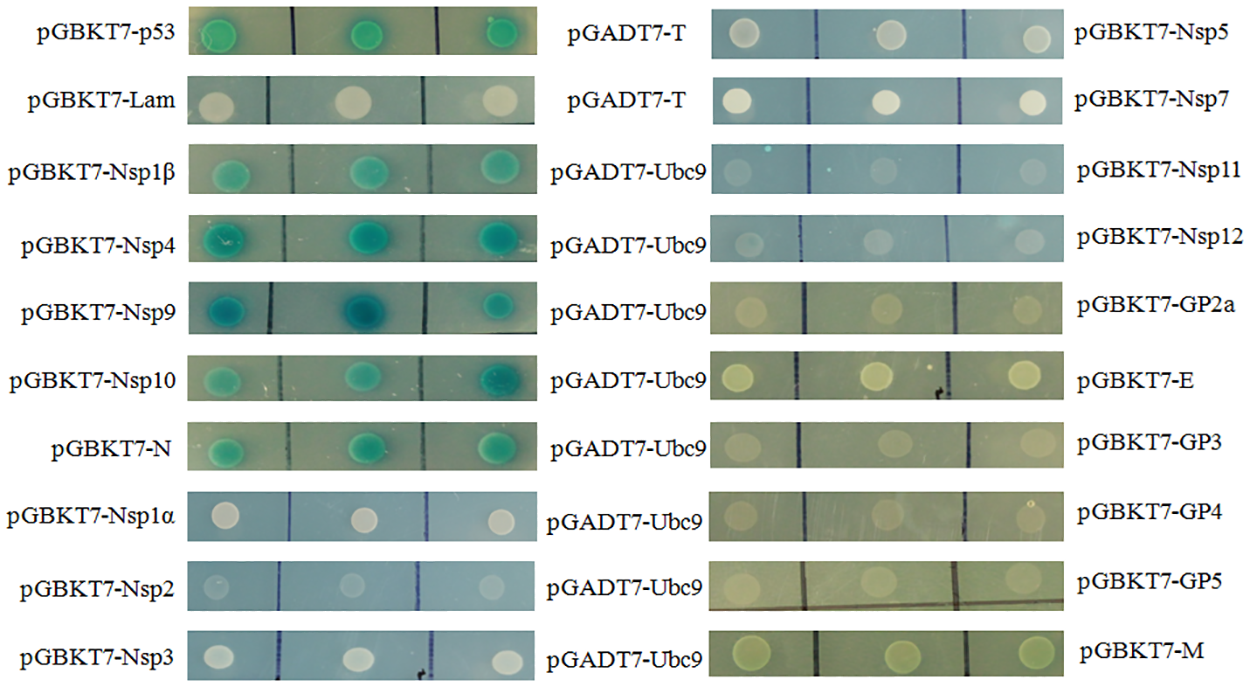
Screening of PRRSV proteins interacting with Ubc9 by yeast two-hybrid. Yeast Y2HGold cells co-transformed with various bait and prey plasmids were selected on QDO/X/ABA plates. Shown are the blue colonies presenting the interaction of PRRSV Nsp1β, Nsp4, Nsp9, Nsp10 and N protein with Ubc9, and white colonies without interaction of the rest of PRRSV proteins with Ubc9. p53 (Gal4 binding domain fused to the murine p53) and T (Gal4 activation domain fused to the SV40 large T-antigen), Lam (Gal4 binding domain fused to nuclear lamina protein) and T were served as positive control and negative control, respectively. The three spots in the same line mean the replicates of one analysis and each spot represents a different colony.

A number of viral proteins have been shown to interact with the SUMO conjugating enzyme Ubc9, which acts as a hub for protein SUMOylation [33]. SUMOylation is not detectable for all the Ubc9-interacting viral proteins during viral invasion, however, some viral proteins are indeed sumoylated, as Ubc9 can directly recognize and sumoylate Lys residues embedded in a SUMOylation consensus motif [39–41]. Therefore, we analyzed the interaction between PRRSV proteins and Ubc9 by a yeast two-hybrid system. The Ubc9 gene was cloned into the prey plasmid (pGADT7) and co-transformed into the yeast Y2HGold cells with the bait (pGBKT7) plasmid expressing each of the nonstructural (except for Nsp2TF, Nsp2N, Nsp6 and Nsp8) and structural proteins of PRRSV JXwn06. The transformed colonies were cultivated on the synthetic selection medium QDO/X/ABA plates and the blue colonies indicated the physical interaction of PRRSV proteins with Ubc9, whereas no colonies growth showed no interaction among the proteins mentioned above. As shown in Fig 1, the Nsp1β, Nsp4, Nsp9, Nsp10 and N protein were shown to interact with Ubc9.

### Identification of the interaction of Ubc9 with Nsp1β, Nsp4, Nsp9, Nsp10 and N protein

**Fig 2:**
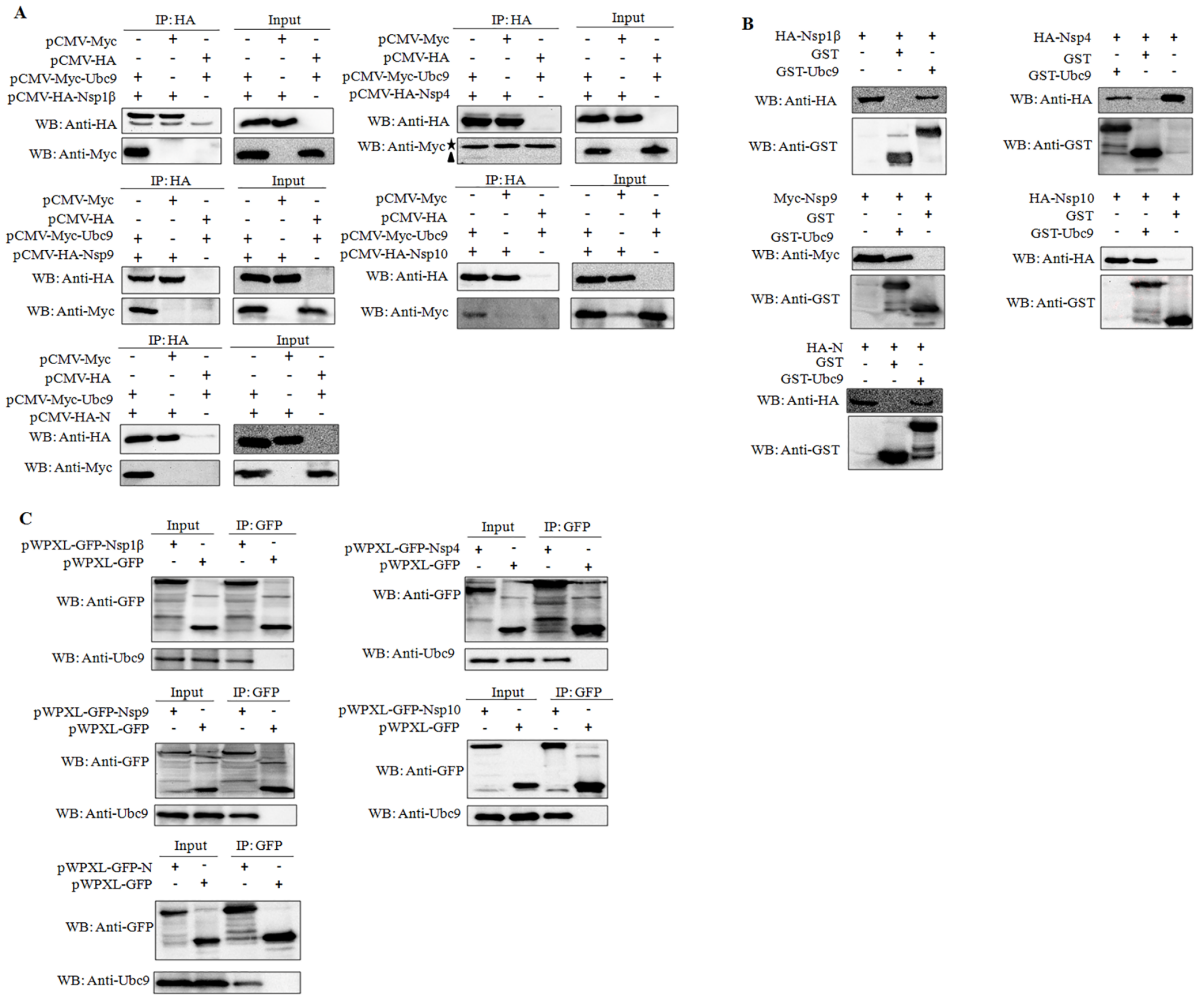
The interaction of PRRSV Nsp1β, Nsp4, Nsp9, Nsp10 and N protein with Ubc9. **(A)** The interaction of Nsp1β, Nsp4, Nsp9, Nps10 and N protein with exogenous Ubc9 by using a Co-IP assay. HEK293 cells were co-transfected with the Myc-Ubc9-expressing plasmid and the HA-Nsp1β-, HA-Nsp4-, HA-Nsp9-, HA-Nsp10- and HA-N-expressing plasmid, respectively. The cell lysates were immunoprecipitated with an anti-HA mAb and probed with anti-HA mAb and anti-Myc PAb. The left panel shows the Co-IP analyses of HA-Nsp1β, HA-Nsp4, HA-Nsp9, HA-Nsp10 and HA-N from cell lysates and the right panel indicates the identification of HA-Nsp1β, HA-Nsp4, HA-Nsp9, HA-Nsp10 and HA-N expressed in cell lysates. The asterisk (★) indicates the IgG light chain band with 26 KDa, and the solid triangle (▲) represents the target protein Myc-Ubc9. **(B)** The interaction of Nsp1β, Nsp4, Nsp9, Nsp10 and N protein with exogenous Ubc9 by using a GST pull-down assay. The cell lysates containing Nsp1β, Nsp4, Nsp9, Nsp10 and N protein individually were pulled down with prokaryotic expressed and purified GST-Ubc9 protein with an anti-GST mAb and probed with anti-HA and anti-GST mAb. **(C)** The interaction of Nsp1β, Nsp4, Nsp9, Nsp10 and N protein with endogenous Ubc9. MARC-145 cells were transduced with the lentiviruses that were expressing GFP, Nsp1β, Nsp4, Nsp9, Nsp10, or N individually. The cell lysates were immunoprecipitated with an anti-GFP mAb and followed by Western blot analysis with anti-Ubc9 and anti-GFP antibodies. The left panel indicates the identification of GFP, Nsp1β-GFP, Nsp4-GFP, Nsp9-GFP, Nsp10-GFP and N-GFP expressed in cell lysates, while the right panel shows the Co-IP analyses of GFP, Nsp1β-GFP, Nsp4-GFP, Nsp9-GFP, Nsp10-GFP and N-GFP from cell lysates.

To further confirm the interactions screened above, the HEK293 cells that were co-expressing each HA-tagged protein (Nsp1β, Nsp4, Nsp9, Nsp10 or N protein) of PRRSV and Myc-Ubc9 were cultured. The lysates of HEK293 cells were then immunoprecipitated using an antibody to HA. The results showed that all of them could be detected by Western blot in the precipitate, indicating that these proteins interact with exogenous Ubc9 in HEK293 cells (Fig 2A). To further verify the binding of these proteins to exogenous Ubc9, these proteins were expressed in HEK293 cells respectively, and their interactions with exogenous Ubc9 were examined using a GST pull-down assay. As shown in Fig 2B, the binding of each viral protein with prokaryotic-expressed Ubc9 was readily detectable in the cells that were expressing Myc-tagged Nsp1β, Nsp4, Nsp9, Nsp10 or N protein. Moreover, MARC-145 cells were transduced with the lentiviruses that were expressing Nsp1β-GFP, Nsp4-GFP, Nsp9-GFP, Nsp10-GFP or N-GFP, respectively, and the cell lysates were immunoprecipitated with an anti-GFP mAb, and were then detected with an anti-Ubc9 PAb by a co-immunoprecipitation (Co-IP) assay. The results showed that endogenous Ubc9 could be probed in the cells that were expressing each viral protein (Fig 2C), indicating that Ubc9 can interact with Nsp1β, Nsp4, Nsp9, Nsp10 or N protein of PRRSV, respectively.

### Nsp1β, Nsp4, Nsp9, Nsp10 and N protein co-localized with Ubc9

**Fig 3:**
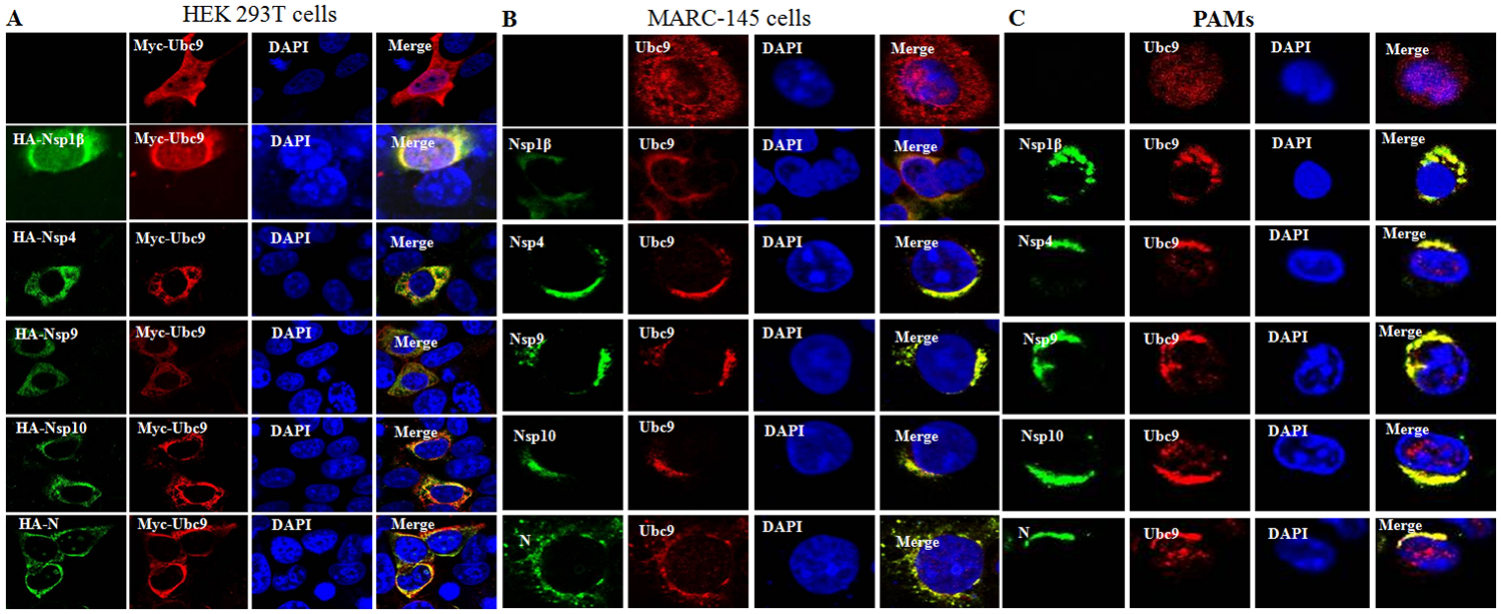
Co-localization of Nsp1β, Nsp4, Nsp9, Nsp10 and N protein with Ubc9. **(A)** Co-localization of Nsp1β, Nsp4, Nsp9, Nsp10 and N protein with exogenous Ubc9 in HEK293 cells. HEK293 cells were co-transfected with pCMV-HA-Nsp1β, pCMV-HA-Nsp4, pCMV-HA-Nsp9, pCMV-HA-Nsp10 and pCMV-HA-N with pCMV-Myc-Ubc9, respectively. The cells were fixed at 36 h post-transfection and processed by immunostaining with a mouse anti-HA mAb or rabbit anti-Myc PAb and TRITC-conjugated goat anti-mouse IgG or FITC-conjugated goat anti-rabbit IgG, and were then examined by confocal microscopy (600×magnification). Nuclei were stained with DAPI. Co-localization of Nsp1β, Nsp4, Nsp9, Nsp10 and N protein with endogenous Ubc9 in mock-infected MARC-145 cells and PRRSV-infected MARC-145 cells **(B)**, mock-infected PAMs and PRRSV-infected PAMs **(C)**. The mock- or PRRSV-infected cells were fixed at 24 h post-infection and processed by immunostaining with a rabbit anti-Ubc9 PAb or mouse anti-Nsp1β, anti-Nsp4, anti-Nsp9, anti-Nsp10 and anti-N mAb, respectively, and then immunostained with TRITC-conjugated goat anti-mouse IgG and FITC-conjugated goat anti-rabbit IgG. Nuclei were stained with DAPI.

To determine the subcellular localization of Ubc9 with the proteins of PRRSV, HEK293 cells were co-transfected with pCMV-Myc-Ubc9 and each pCMV that was expressing HA-tagged viral protein (Nsp1β, Nsp4, Nsp9, Nsp10 or N). The cells were then examined by confocal imaging. The results indicated that Ubc9 could co-localize with each of the nonstructural proteins in the cytoplasm, while Ubc9 co-localized with N protein mainly in the cytoplasm and a few in the nucleus (Fig 3A). To determine whether endogenous Ubc9 localizes with the viral proteins in PRRSV-infected cells, MARC-145 cells and PAMs were infected with PRRSV JXwn06, and an indirect immunofluorescence assay (IFA) was then performed. Consistent with the above observations, endogenous Ubc9 was also shown to co-localize with the viral proteins in the cytoplasm of PRRSV-infected cells (Fig 3B and 3C). These results clearly reveal that Ubc9 interacts with the Nsp1β, Nsp4, Nsp9, Nsp10 or N protein in the cytoplasm of PRRSV-infected cells.

### Effect of Ubc9 on PRRSV replication

**Fig 4:**
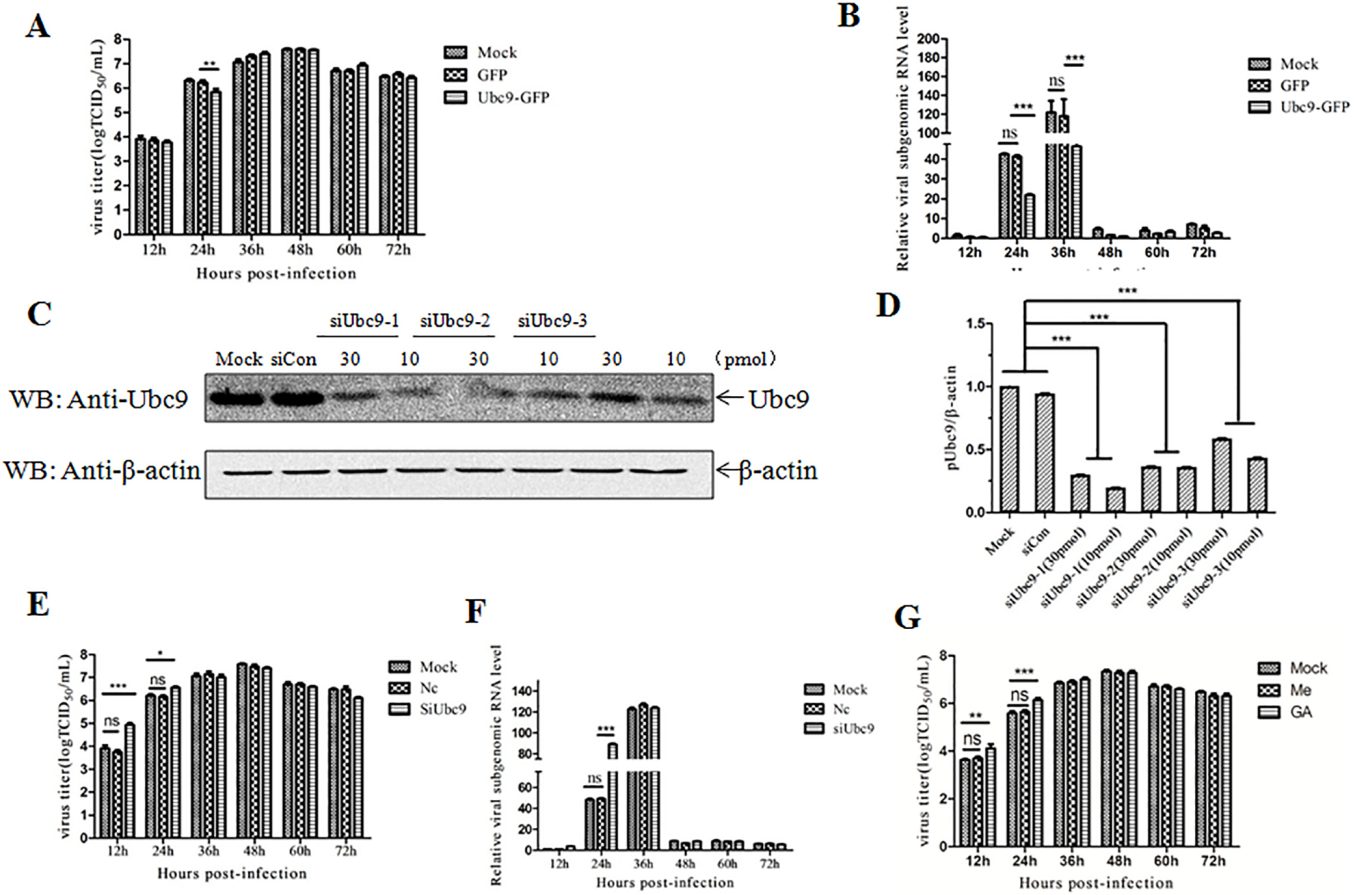
Inhibition of PRRSV JXwn06 replication by Ubc9. **(A)** PRRSV titers in Ubc9-overexpressed MARC-145 cells. MARC-145 cells were transduced with the lentiviruses that were expressing GFP and Ubc9, respectively. The cells were infected with PRRSV JXwn06 at MOI of 0.01 at 24 h post-transduction, and the virus titers were then assayed by a microtitration infectivity assay at the indicated time points post-infection. Data are shown as means ± SD of three independent experiments (**p<0.01). **(B)** PRRSV RNA replication in Ubc9-overexpressing MARC-145 cells. MARC-145 cells were transduced with the lentiviruses that were expressing GFP and Ubc9, respectively. The cells were infected with PRRSV JXwn06 at MOI of 0.01 at 24 h post-transduction and collected at the indicated time points post-infection. The total cellular RNA was extracted and the mRNA levels of PRRSV N gene were determined by quantitative RT-PCR. Data are shown as means ± SD of three independent experiments (***p<0.001; ns, no significant). **(C)** SiRNA-mediated knockdown of endogenous Ubc9. MARC-145 cells were transfected with siRNA (siUbc9-1, siUbc9-2, siUbc9-3) and control siRNA (siCon). The cells were harvested at 48 h post-transfection and the cell lysates were probed with an anti-ubc9 antibody. The optical density ratios of Ubc9/β-actin in Ubc9 gene-silenced MARC-145 cells are shown with graphs. Data are shown as means ± SD of three independent experiments (***p<0.001). **(D)** The optical density ratios of Ubc9/β-actin in Ubc9 gene-silenced MARC-145 cells are shown with graphs. Data are shown as means ± SD of three independent experiments (***p<0.001). **(E)** PRRSV titers in Ubc9 gene-silenced MARC-145 cells. MARC-145 cells transfected with the siRNA (siUbc9-1) or control siRNA (siCon) for 48 h were infected with PRRSV JXwn06 at MOI of 0.01, and the virus titers were examined at the indicated time points post-infection. Data are shown as means ± SD of three independent experiments (*p<0.05; ***p<0.001; ns, no significant). **(F)** PRRSV RNA replication in Ubc9-silenced MARC-145 cells. MARC-145 cells transfected with the siRNA (siUbc9-1) or control siRNA (siCon) for 48 h were infected with PRRSV JXwn06 at MOI of 0.01 and collected at the indicated time points post-infection. The total cellular RNA was extracted and the mRNA levels of PRRSV N gene were determined by quantitative RT-PCR. Data are shown as means ± SD of three independent experiments (***p<0.001; ns, no significant). **(G)** PRRSV growth in the MARC-145 cells treated with GA. Data are shown as means ± SD of three independent experiments (***p<0.001; **p<0.01; ns, no significant).

As several Ubc9-interacting proteins of PRRSV are involved in the genomic transcription and replication of PRRSV, it is proposed that Ubc9 is involved in the life cycle of PRRSV. The effect of Ubc9 on the replication of PRRSV was investigated in Ubc9-overexpressing MARC-145 cells. Quantitative RT-PCR analysis showed that the viral mRNA levels of ORF7 gene in the Ubc9-overexpressing cells were significantly decreased in the early phase of PRRSV infection (Fig 4A), suggesting the negative role of Ubc9 in the genomic RNA synthesis. However, compared with the control group, the virus titers had no significant change in the Ubc9-overexpressing cells (Fig 4B). To further confirm the effect of Ubc9, MARC-145 cells were individually transfected with three siRNAs specific for the Ubc9 gene. Western blot analysis showed that the Ubc9 level in MARC-145 cells was effectively down-regulated in Ubc9-specific siRNA, but not controls siRNA-transfected MARC-145 cells (Fig 4C, 4D and S1 Fig). Knockdown of Ubc9 in MARC-145 cells could significantly promote the genomic RNA synthesis of PRRSV and increased viral titers in the early phase of infection (Fig 4E and 4F), further indicating that Ubc9 plays a role in the inhibition of PRRSV replication in the early phase of infection. Finally, we analyzed whether the SUMOylation inhibitor can enhance the replication of PRRSV. The data showed that the replication of PRRSV was enhanced in the MARC-145 cells treated with GA at 12 h and 24 h post-infection (Fig 4G). These findings indicate that the interaction of cellular Ubc9 with the viral proteins is beneficial for host cells to restrict the replication of PRRSV in the early phase of infection.

### SUMOylation status of N protein during PRRSV infection

**Fig 5:**
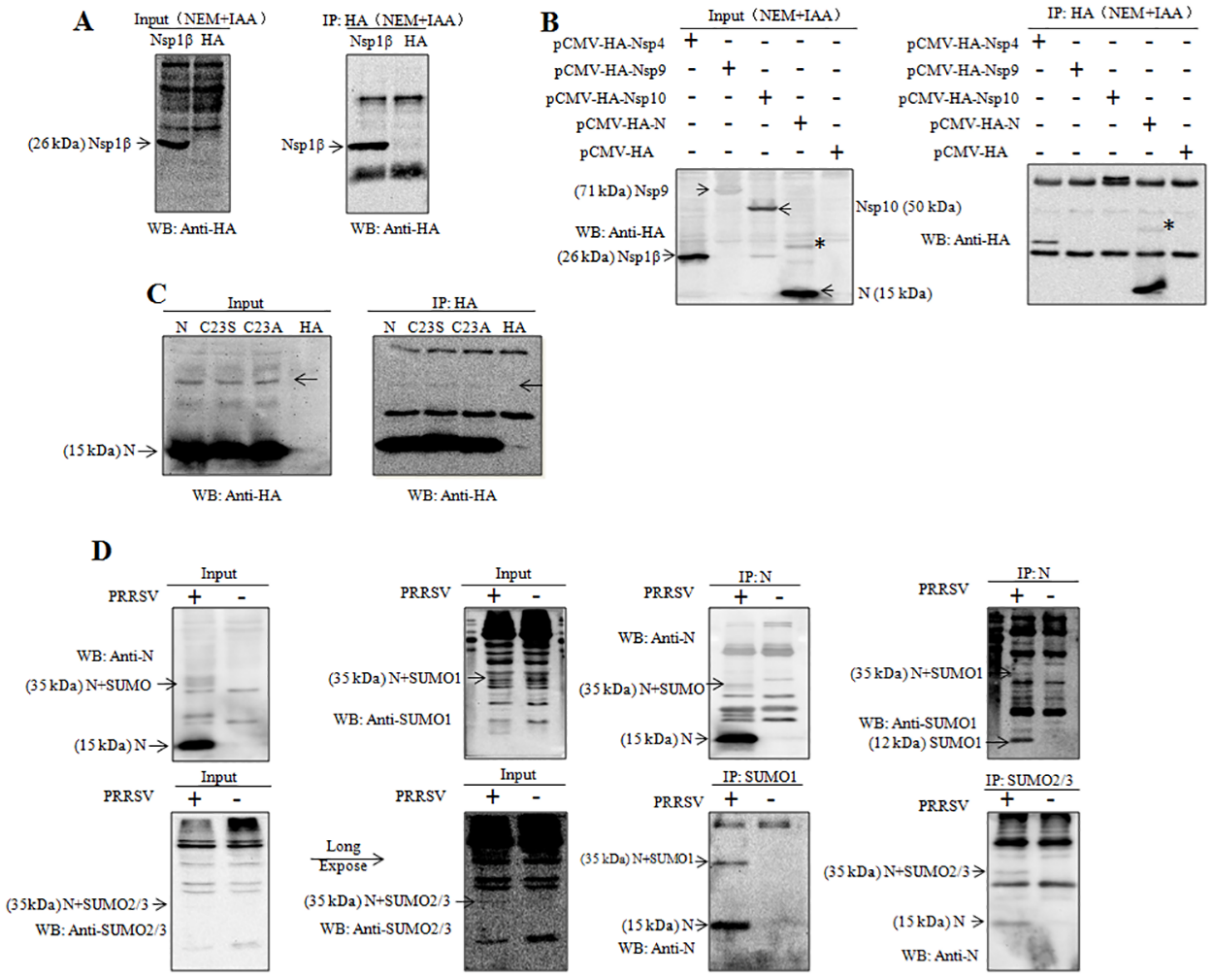
SUMOylation of PRRSV N protein. **(A and B)** The expression of Nsp1β, Nsp4, Nsp9, Nsp10 and N proteins in HEK293 cells using a Co-IP assay. HEK293 cells were transfected with pCMV-HA-Nsp1β, pCMV-HA-Nsp4, pCMV-HA-Nsp9, pCMV-HA-Nsp10 and pCMV-HA-N, separately. The cell lysates were immunoprecipitated with an anti-HA mAb and probed with an anti-HA mAb. The left panel indicates the identification of HA-Nsp1β, HA-Nsp4, HA-Nsp9, HA-Nsp10 and HA-N expressed in cell lysates and the right panel shows the Co-IP analyses of HA-Nsp1β, HA-Nsp4, HA-Nsp9, HA-Nsp10 and HA-N from cell lysates. **(C)** The expression of N and mutated N proteins (C23S and C23A) in HEK293 cells by using a Co-IP assay. HEK293 cells were transfected with pCMV-HA-N, pCMV-HA-N (C23S), pCMV-HA-N (C23A), separately. The cell lysates were immunoprecipitated with an anti-HA mAb and probed with anti-HA mAb. The left panel indicated the identification of HA-N, HA-N (C23S) and HA-N (C23A) expressed in cell lysates and the right panel showed the Co-IP analyses of HA-N, HA-N (C23S) and HA-N (C23A) from cell lysates. **(D)** The expression of N protein in MARC-145 cells following PRRSV infection using a Co-IP assay. MARC-145 cells were infected with PRRSV JXwn06 at a MOI of 0.1. At 36 h the cell lysates were immunoprecipitated with an anti-N mAb, anti-SUMO1 or anti-SUMO2/3 mAb and probed with these mAb, separately.

To verify the SUMOylation property of the proteins of PRRSV, HEK293 cells were transfected with the Nsp1β-, Nsp4-, Nsp9-, Nsp10- or N-expressing plasmid, respectively. The cell lysates were then detected by Western blot using an anti-HA antibody. Intriguingly, several bands could be observed in the N-expressing HEK293 cells, and the single band with nearly 35 kDa was confirmed to be the modified form of N protein through Co-IP assay. Because the size of sumoylated protein is usually considered to be 20 kDa larger than the unmodified one by performing a SDS-PAGE, the band with a size of nearly 35 kDa which was 20 kDa larger than the normal N protein, was postulated to be the SUMOylation form of N protein (Fig 5A, 5B and S2 Fig). In order to rule out the possibility of homodimerized form of N protein, N protein mutants with the amino acid mutation at the position 23 from cysteine to serine (C23S) and from cysteine to alanine (C23A) were generated. The results showed that the mutations of cysteine at this position completely blocked the homodimerization of N protein, while the band with a size of 35 kDa remained to be detected by using an anti-HA antibody, indicating that the protein band was very likely generated by SUMOylation, not by homodimerization (Fig 5C and S3 Fig). There is a great possibility that overexpression of exogenous protein in HEK293 cells leads to the misfolding of protein and exposure of internal amino acid residues to the interface, then further resulting in protein modifications that may not be detected in the natural status. Therefore, to further confirm whether N protein can be sumoylated during PRRSV infection, MARC-145 cells were infected with PRRSV JXwn06 and Co-IP assay was performed using a mAb against N protein of PRRSV, SUMO1 and SUMO2/3. When performing a Co-IP assay with a mAb to PRRSV N protein, the band with a size of 35 kDa could be detected with not only a mAb to N protein, but also a mAb to SUMO1 and SUMO2/3 (Fig 5D and S4 Fig), indicating the SUMOylation of PRRSV N protein is executed by either SUMO1 or SUMO2/3 during PRRSV infection. Moreover, the band with a size of 35 kDa could also be detected using a mAb against PRRSV N protein when Co-IP assay was performed with either antibody to SUMO1 or SUMO2/3 (Fig 5D), further demonstrating the SUMOylation status of PRRSV N protein during PRRSV infection. Of note, when Co-IP assay was performed with either SUMO1 or SUMO2/3 antibodies, both sumoylated and the normal form of N proteins could be detected using a mAb to N protein, showing that PRRSV N protein can also interact directly with both SUMO1 and SUMO2/3.

## Discussion

Among the numerous post-translational modifications occurring in cells, SUMOylation plays pivotal role in controlling the function of a plethora of proteins and biological processes. In consequence of its central regulatory role, the SUMOylation pathway is widely exploited by viruses, whose proteins can either modify and/or be modified by the SUMOylation system with various outcomes [36–38].

As Ubc9 directly recognize the SUMOylation acceptor site embedded in a SUMOylation consensus motif and mediate SUMO conjugation [39–41], in our study, we searched for the proteins of PRRSV which interact with Ubc9 using a yeast two-hybrid system. Finally, the Nsp1β, Nsp4, Nsp9, Nsp10 and N protein of PRRSV were screened out and their interactions with Ubc9 were confirmed. A study on type 2 dengue virus (DV-2) showed that the overexpression of Ubc9 can reduce the viral plaque formation in mammalian cells [39–41], while another study indicated that, during the late stages of HIV-1 replication, Ubc9 is crucial cellular protein to produce infectious particles [51]. Our present studies demonstrated that, in MARC-145 cells, the overexpression of Ubc9 could inhibit the genomic replication of PRRSV in the early phase of infection, whereas the siRNA-mediated knockdown of Ubc9 could significantly promote the genomic RNA synthesis of PRRSV and increased viral titers. These results imply that the cellular Ubc9 plays a role in restricting the replication of PRRSV via its interaction with viral proteins in the early phase of infection. Further study will be required to focus on the molecular mechanism of Ubc9 involvement in PRRSV replication.

Ubc9, that functions as a hub for protein SUMOylation, may not only affect the SUMOylation of the Nsp4, Nsp9, Nsp10 and N protein via direct interaction, but also be hijacked by its partner proteins which will further extend the impact of SUMOylation on a multitude of downstream effector pathways. Taking the latent membrane protein 1 (LMP1) of Epstein-Barr virus (EBV) as an example, the interaction between Ubc9 and the C-terminal-activating region 3 of LMP1 can promote the SUMOylation of downstream targets, and affects cellular migration [52]. Moreover, Ubc9 also plays a dual role independent of its SUMO-conjugase manner [53–60], further expanding the probably consequences of their interactions.

For the purpose of searching for SUMOylation of PRRSV proteins, we focused on these five proteins and identified the SUMOylation of N protein. Because of the lower transfection efficiency of these proteins and the lower sensitivity of this method, we could not exclude the possibility of Nsp1β, Nsp4, Nsp9 and Nsp10 to be sumoylated. The most classical method used to detect protein SUMOylation *in vivo* is based on establishing cells lines that express 6×His-SUMO stably at a level similar to the endogenous protein [61]. In order to enrichment the endogenous SUMO targets, an effective strategy has been evolved based on the use of anti-SUMO immunoprecipitation and peptide elution [62]. With the higher sensitive and accuracy methods mentioned above, we may go further to identify whether other PRRSV proteins are potential SUMO-targets.

SUMO has been found covalently attached to numerous host proteins that participate in multiple cellular pathways, including transcription, DNA repair, nuclear transport, signal transduction and the cell cycle [27, 35]. Besides the host proteins, it has been proposed that a large number of viral proteins are SUMO-targets and regulation of these proteins by SUMOylation system is essential for the life cycle of viruses [36–38]. SUMOylation has been shown to promote enterovirus 3C protein ubiquitination for degradation and attenuate its cleavage activity of protease *in vitro*, correlating with a reduction in viral replication and apoptosis [63]. BZLF1, a viral transactivator that determines the transition from latent to lytic phases of Epstein-Barr virus life cycle, is post-translationally modified by both SUMO1 and SUMO2/3 at lysine 12. The K12R mutant of BZLF1, which no longer becomes sumoylated, diminishes its ability to disrupt PML-NBs and stimulates gene expression and reactivation of latent EBV to a higher level than the wild-type protein [64–67]. Adenovirus type 5 E1B 55K is a classical viral SUMO E3 ligase that can be sumoylated on Lys104. Substitution of this residue affects its functions, including interaction with PML, the ability to induce degradation of the PML NB-associated DAXX-ATRX chromatin-remodelling complex, and modulation of host p53 function and induction of cellular transformation [68–71]. The matrix protein VP40 of Ebola virus (EBOV) has been confirmed to be sumoylated, which not only maintains the stability of VP40, but also participates in the formation of virus-like particles (VLPs) [72]. The SUMOylation of influenza A virus NS1 has been shown to enhance the NS1 stability and its Ubc9-mediated SUMOylation is targeted for SUMO1, leading to promote the rapid growth of influenza A virus [73]. The SUMOylation of Matrix protein M1 is required for the formation of M1-vRNP complex, and further affects the assembly and morphogenesis of Influenza A virus, while sumoylated nucleoprotein is essential for its intracellular trafficking and virus growth [74]. Thus, SUMOylation of N protein suggests that it may be involved in the biological functions of N protein in the biogenesis and replication of PRRSV. Further researches are required to explore the effect of N protein SUMOylation on the replication and transcription of PRRSV genome.

Nucleocapsid (N) protein, one of the most abundant viral proteins produced within the cells during PRRSV invasion, is highly immunogenic in pigs and has important roles in PRRSV replication and immune evasion. Of note, recent studies indicated that the N-N non-covalent domain of the nucleocapsid protein of type 2 PRRSV enhances the induction of interleukin-10 (IL-10) and expression of regulatory T-lymphocytes (Tregs), and the 15N and 46R residues in PRRSV N protein are critical for Tregs proliferation [75–77]. Although PRRSV replicates in the cytoplasm, as is the case of most RNA viruses, N protein is found in both cytoplasmic and nuclear compartments during infection [78]. It has been postulated that the nuclear localization of the N protein may participates in the regulation of several host cell processes, including regulation of the cell cycle, apoptosis and the induction of antiviral responses. Peptide sequence analysis of the PRRSV N protein has identified two nuclear localization signal (NLS) motifs at position 10–13 and 41–47, called NLS-1 and NLS-2, respectively. While NLS-2 is a functional domain that affects N protein nuclear localization during viral infection and plasmid transfection, NLS-1, the cryptic one, maintains the nuclear localization of NLS-2-null mutants when removing of eight amino acids from the C-terminal end within the conformation-determining region [79]. Of note, PRRSV with mutation of the NLS-2 is shown to induce lower viremia, while higher neutralizing antibody level than the wild-type virus [80–82]. In addition, N protein contains a nuclear localization sequence (NoLS) and a possible nuclear export signal (NES) and all of these domains determine the nucleo-cytoplasmic shuttling properties of PRRSV N protein [79, 83]. SUMO addition, either covalent conjugation or non-covalent interaction, may lead to changes in the stability, localization and function of target protein, and the nuclear localization of several substrates are regulated by SUMOylation [74, 84–86]. Furthermore, Ubc9 that acts independent of its SUMO conjugation function, can interact with a nuclear localization signal motif (QKRKKRR), similar to N protein NLS-1 (QKRKKGN), at the N terminus of Vsx-1 protein, and mediate its nuclear localization [60]. Therefore, it will be meaningful to investigate whether SUMO modification or Ubc9 interaction is essential for regulation of N protein trafficking.

In summary, our results revealed that SUMO E2 conjugase Ubc9 could interact with the Nsp1β, Nsp4, Nsp9, Nsp10 and N protein of PRRSV, and the N protein could be sumoylated as a bona fide target of SUMOylation. To our knowledge, this is the first study indicating the interplay between PRRSV and SUMOylation. Our current study provides a novel insight for understanding the replication and pathogenesis of PRRSV.

## Supporting information

S1 Fig The original picture of Fig 4C. (PDF)

S2 Fig The full blot images of Fig 5A and 5B. (PDF)

S3 Fig The original pictures of Fig 5C. (PDF)

S4 Fig The original pictures of Fig 5D. (PDF)
