# Supplementary figures and images for: Interaction of porcine reproductive and respiratory syndrome virus proteins with SUMO-conjugating enzyme reveals the SUMOylation of nucleocapsid protein
Source: PLoS One. 2017 Dec 13;12(12):e0189191. doi: 10.1371/journal.pone.0189191 (PMC5728522; doi:10.1371/journal.pone.0189191)

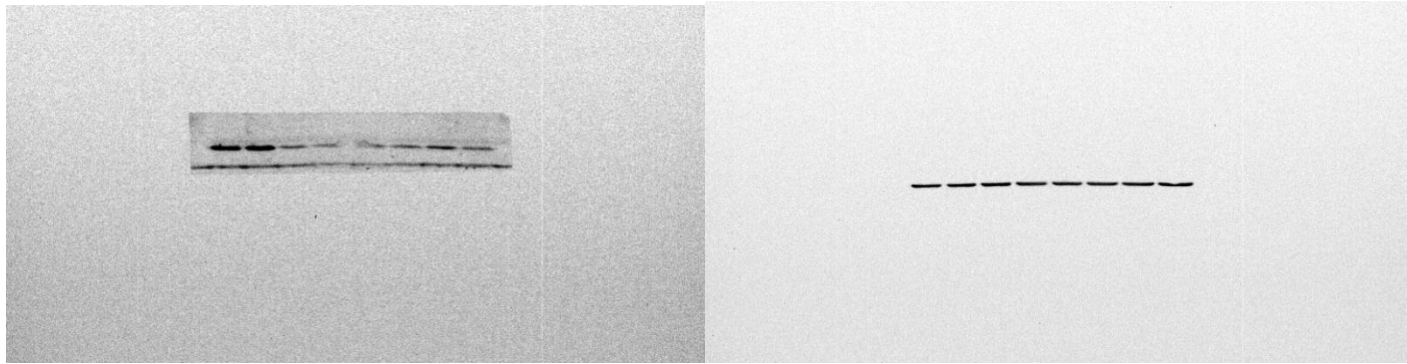

**S1 Fig.** The original picture of Fig 4C

Supplement: S1 Fig — (PDF) [file pone.0189191.s001.pdf]

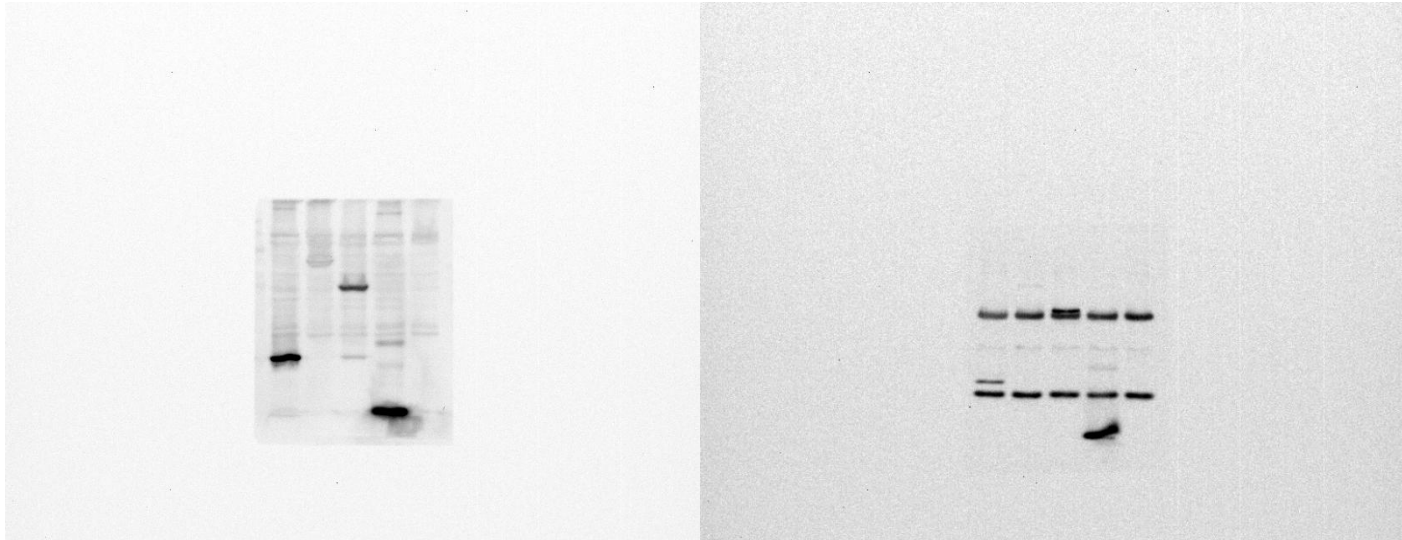

**S2 Fig.** The full blot images of Fig 5A and 5B

Supplement: S2 Fig — (PDF) [file pone.0189191.s002.pdf]

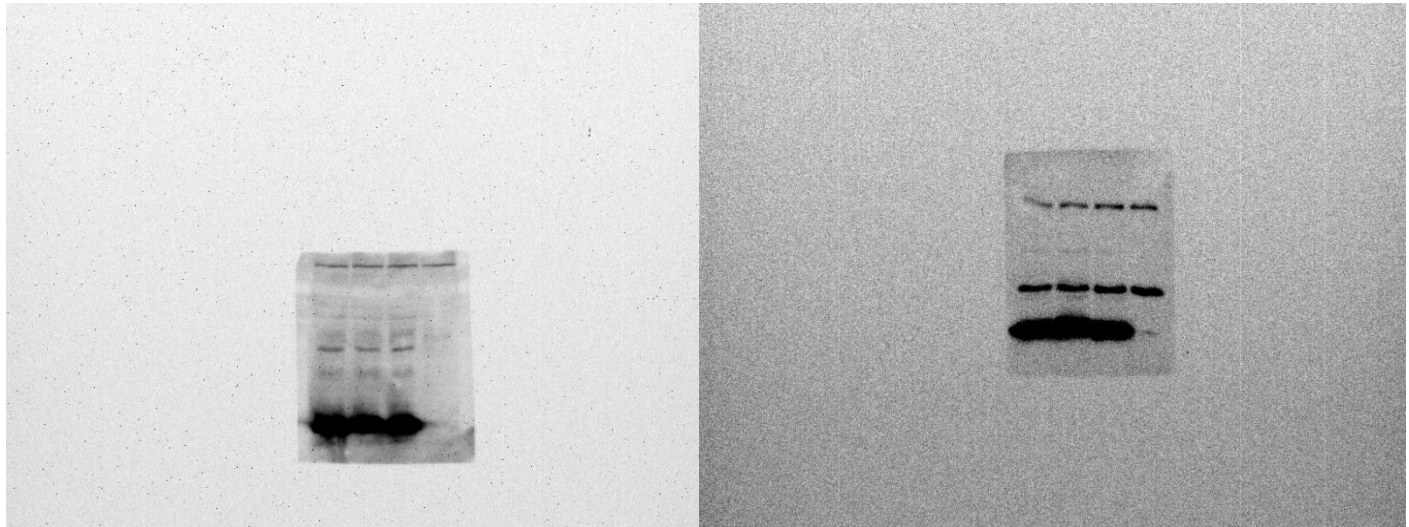

**S3 Fig.** The original pictures of Fig 5C

**S4 Fig**

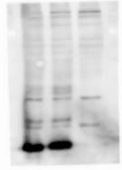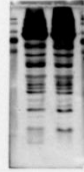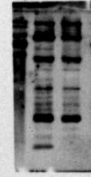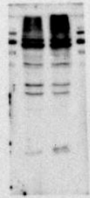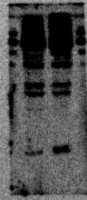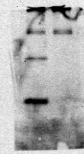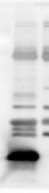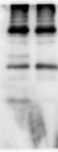

Supplement: S3 Fig — (PDF) [file pone.0189191.s003.pdf]

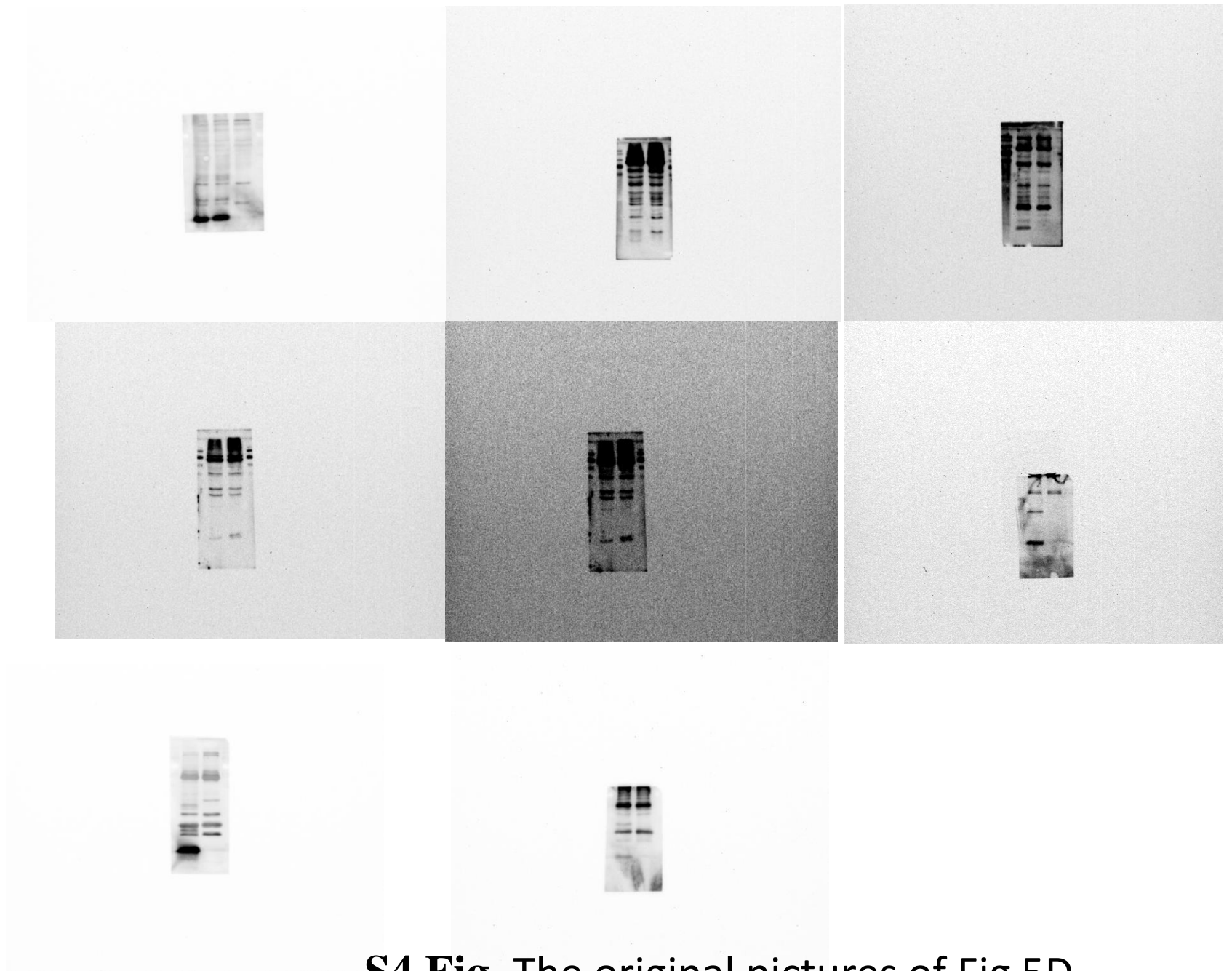

**S4 Fig.** The original pictures of Fig 5D

Supplement: S4 Fig — (PDF) [file pone.0189191.s004.pdf]
